# Comparison of clinical outcomes and second-look arthroscopic evaluations between anterior cruciate ligament anteromedial bundle augmentation and single-bundle anterior cruciate ligament reconstruction

**DOI:** 10.1186/s43019-020-00058-z

**Published:** 2020-08-31

**Authors:** Gil Yeong Ahn, Tae Hun Lee, Kyung Jin Lee, Sangwon Woo

**Affiliations:** Department of Orthopedic Surgery, Pohang St. Mary’s Hospital, Daejamdong-gil 17, Nam-Gu, Pohang, Kyung-Buk 37661 South Korea

**Keywords:** Knee, Anterior cruciate ligament, Augmentation, Reconstruction, Second-look arthroscopy, Synovial coverage

## Abstract

**Subject:**

This study compared clinical outcomes and second-look arthroscopic evaluations between anterior cruciate ligament (ACL) anteromedial (AM) bundle augmentation and single-bundle ACL reconstruction.

**Purpose:**

We compared the clinical results and the second-look arthroscopic findings between (1) single-bundle ACL reconstruction in complete rupture and (2) ACL AM bundle augmentation in isolated AM bundle rupture.

**Materials and methods:**

Two groups of patients underwent ACL surgery from January 2013 to December 2018. Group 1, who had 64 cases of single-bundle ACL reconstruction with second-look arthroscopy, and Group 2, who had 21 cases of AM bundle augmentation of ACL with second-look arthroscopy, were targeted. We evaluated and compared the clinical results (Lysholm score, Tegner activity score, Lachman test, and pivot-shift test) and synovialization at second-look arthroscopy before the operation and in the final follow-up period, between Group 1 and Group 2.

**Results:**

The Lysholm score (*p* = 0.96) and Tegner activity score (*p* = 0.351) at final follow-up (mean 27.1 months) were 78.3 and 7.2 in Group 1 and 89.1 and 8.1 in Group 2, respectively. The Lachman test (*p* = 0.074) and pivot-shift test (*p* = 0.031) results at final follow-up were improved; however, there was no statistical significance. Second-look arthroscopy showed that percentages of synovialization area of grafted tendon at mean 15.6 months follow-up were 61.4% and 93.1% in Group 1 and Group 2, respectively (*p* = 0.008). The synovial coverage in Group 2 was higher than in Group 1.

**Conclusion:**

The AM bundle augmentation for ACL injury in which the posterolateral bundle was preserved showed better clinical scores and synovial coverage than single-bundle ACL reconstruction for complete ACL rupture.

**Level of evidence:**

The level of evidence is Level III, retrospective with case series.

## Introduction

For successful results of anterior cruciate ligament (ACL) reconstruction, not only good mechanical stability but also functional recovery of proprioceptive sensation and revascularization of graft tendon are needed [1–3]. Recently, the use of remnant preservation procedures is increasing if remnant ligament tissue remains during ACL reconstruction [1–5]. In particular, many studies reported good results of anteromedial (AM) or posterolateral (PL) bundle augmentation operation when the ACL was partially ruptured [6–12].

During ACL reconstruction, the remnant preservation technique is well known to help maintain stable reflex function around the knee joint muscles due to preservation of the mechanoreceptors [2, 3, 13] and to aid in synovialization because of blood vessel regeneration to the graft tendon [8, 14–17].

To preserve remnant tissue during ACL reconstruction, two methods have been introduced. One is preservation of the remnant of the torn ACL at the site of tibial attachment [2, 3, 5, 15, 16], and the other is selective bundle augmentation surgery of the AM bundle or the PL bundle with preservation of the intact bundle [6, 8–11, 18, 19]. There are numerous studies on preservation of the remnant of torn ACL at the tibial attachment site, but studies on selective bundle augmentation of the AM bundle or PL bundle with preservation of the intact bundle are limited.

It is expected that clinical results and second-look arthroscopic findings will be different between single-bundle ACL reconstruction in complete rupture of ACL and AM bundle augmentations when the AM bundle is ruptured with an intact PL bundle [8, 9, 19]. Hence, we compared and analyzed these two different treatments using clinical evaluation, stability tests, and second-look arthroscopic examination.

## Materials and methods

Protocol approval was obtained from the International Review Board of Pohang St. Mary’s Hospital (IRB No. 0749-191107-HR-041-01). The study was performed in accordance with the ethical standards established in the 1964 Declaration of Helsinki and its later amendments. The informed consent requirement was waived.

### Patient selection and study design

**Fig. 1:**
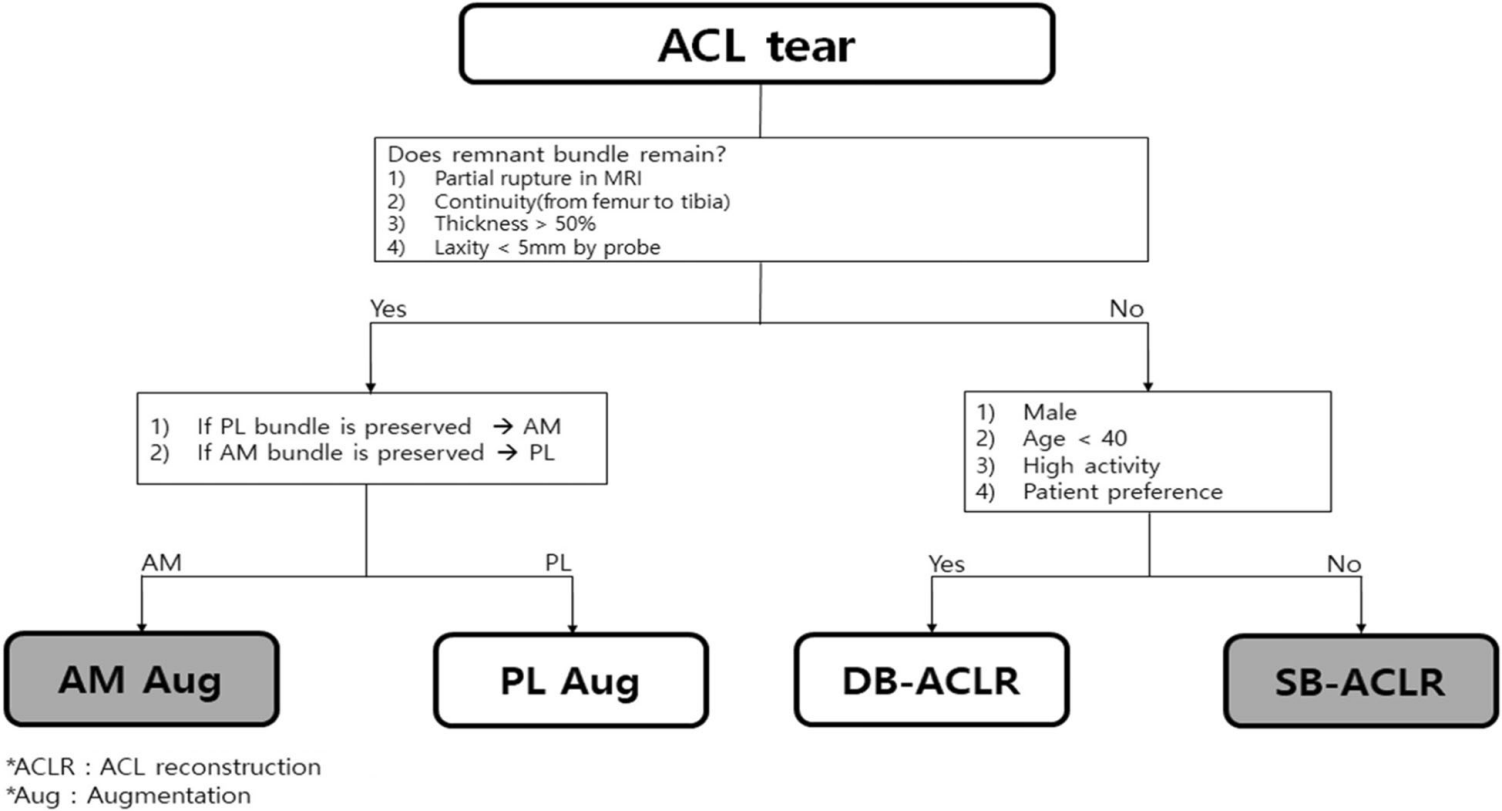
Indications for surgical methods

We retrospectively reviewed the medical records of the patients who underwent primary ACL reconstruction from January 2013 to December 2018. All patients had a magnetic resonance imaging (MRI) examination. If a patient met the following remnant criteria: (1) partial rupture as shown by MRI, (2) continuity from femur to tibia, (3) thickness of ACL of more than 50% of intact of ACL, and (4) laxity of remnant ACL of less than 5 mm as shown by arthroscopy), that patient underwent augmentation surgery for symptomatic ACL partial rupture. If the AM bundle was preserved, the patient was treated with PL augmentation; if the PL bundle was preserved, the patient was treated with AM augmentation. If a patient had no remnant criteria, that patient underwent ACL reconstruction surgery. If a patient met the following double-bundle criteria: (1) male, (2) age less than 40, (3) high activity, and (4) patient preference for double-bundle reconstruction, that patient underwent double-bundle ACL reconstruction, and if not, the patient underwent single-bundle ACL reconstruction (Fig. 1).

Eighty-four cases of double-bundle ACL reconstruction and 10 cases of patients who had an associated injury such as femur or tibia fracture, medial collateral ligament rupture, or posterior cruciate ligament injury were excluded, and six cases of patients with PL bundle augmentation were also excluded, because the number of patients who had PL augmentation was so small and there could be different results for the effect. The number of single-bundle ACL reconstruction cases in complete ACL rupture was 140, and the number of AM bundle augmentation cases in partial rupture of ACL was 88. During the study period, a total of 85 cases were able to be followed up. All 85 patients underwent second-look arthroscopy. Among these patients, there were 64 with primary single-bundle ACL reconstruction in complete rupture of ACL (Group 1) and 21 with selective AM bundle augmentation in partial rupture of ACL (Group 2). RigidFix™ (Mitek, Johnson & Johnson, Raynham, MA, USA) with auto hamstring tendons—four strands of gracilis and semitendinosus tendons—was used in Group 1. RigidFix™ with four strands of auto hamstring tendons for AM bundle augmentations was used in Group 2.

All of the patients underwent surgery within 4 months after injury, and we were able to check the condition of the graft through second-look arthroscopy during removal of the tibial anchor screw after 1–2 years of reconstruction surgery.

The gender of the patients was 59 men and 26 women, with an average age of 34.1 years (Table 1). Sports injury was the most common cause of the damage with 42 cases, followed by 17 cases of direct trauma, 12 cases of traffic accidents, 8 cases of falls, and 6 others. Types of combined injury were medial meniscus tear (37 cases), medial collateral ligament injury (21 cases), lateral meniscus tear (17 cases), minor fracture around the knee joint (5 cases), and posterior cruciate ligament sprain (4 cases) (Table 2). There were 84 total items of combined injuries among 65 patients who had combined injuries. Cases of complete rupture of medial collateral ligament and major fracture of femur or tibia were excluded.

**Table 1:** Demographics of study group

	Group 1 (SB group)	Group 2 (Aug group)	Total	*p* value
Case	64	21	85	0.632
Male:Female	40:24	19:2	59:26	0.381
Age (years)	34.6 (18–58)	32.4 (17–56)	34.1 (17–58)	0.523
Trauma to op. interval (days)	78.1 (7–252)	85.2 (12–300)	78.8 (7–300)	0.374
Interval to 2nd-look A/S (months)	15.4 (11.6–23.3)	16.2 (11.7–35.0)	15.6 (11.6–35.0)	0.283
Follow-up (months)	26.7 (19.2–36.5)	28.3 (20.3–38.0)	27.1 (19.2–38.0)	0.247

Values for age, trauma to op. interval, interval to second-look arthroscopy, and follow-up were described as mean (ranges)

*SB* single bundle, *Aug* anteromedial bundle augmentation, *A/S* arthroscopic surgery

**Table 2:** Combined injuries

Type	Group 1	Group 2	Total	Treatment	
MM tear	29	8	37 (44.0%)	Meniscectomy	19
				Repair	11
				Conservative treatment	7
MCL injury	15	6	21 (25.0%)	Conservative treatment	21
LM tear	12	5	17 (20.2%)	Meniscectomy	11
				Repair	6
Minor fracture	4	1	5 (5.9%)	Conservative treatment	5
PCL sprain	3	1	4 (4.8%)	Conservative treatment	4

Of the total 65 patients, there were 84 cases of associated injury

Minor fracture is avulsion fracture or linear fracture around knee joint

*MM* medial meniscus, *MCL* medial collateral ligament, *LM* lateral meniscus tear, *PCL* posterior cruciate ligament

### Surgical technique

**Fig. 2:**
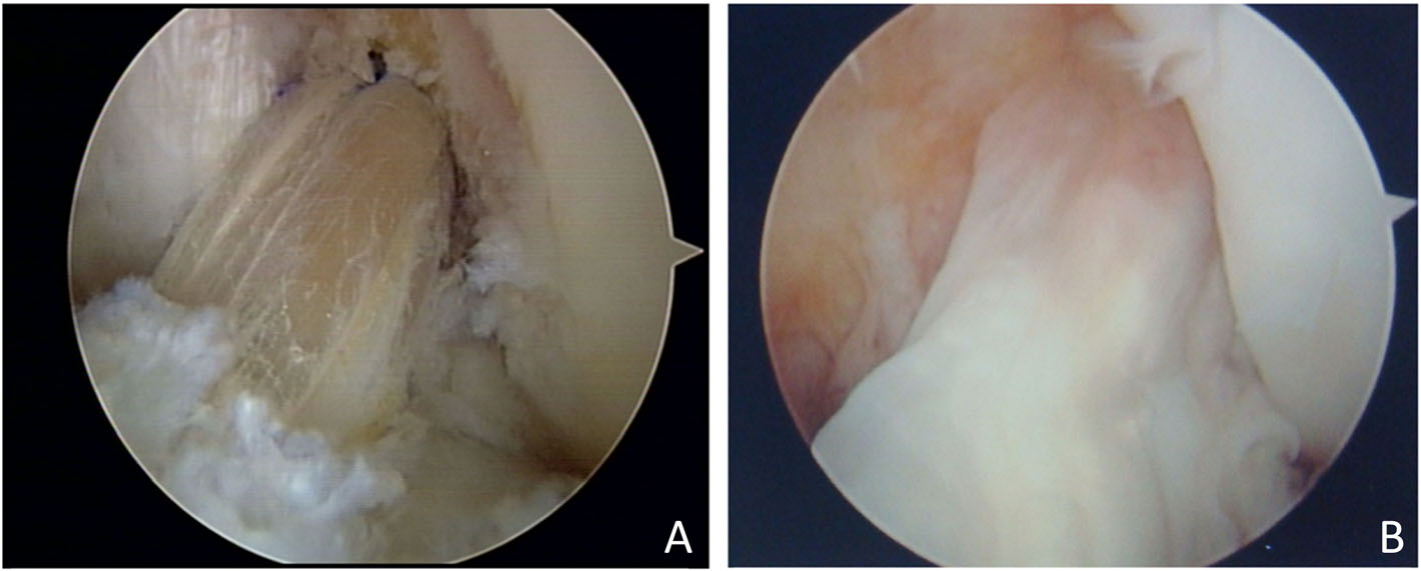
**a** Arthroscopic findings show single-bundle ACL reconstruction using a hamstring autograft with the tibial side remnant preservation technique. **b** The second-look arthroscopic finding shows large extent of synovial coverage on the graft

**Fig. 3:**
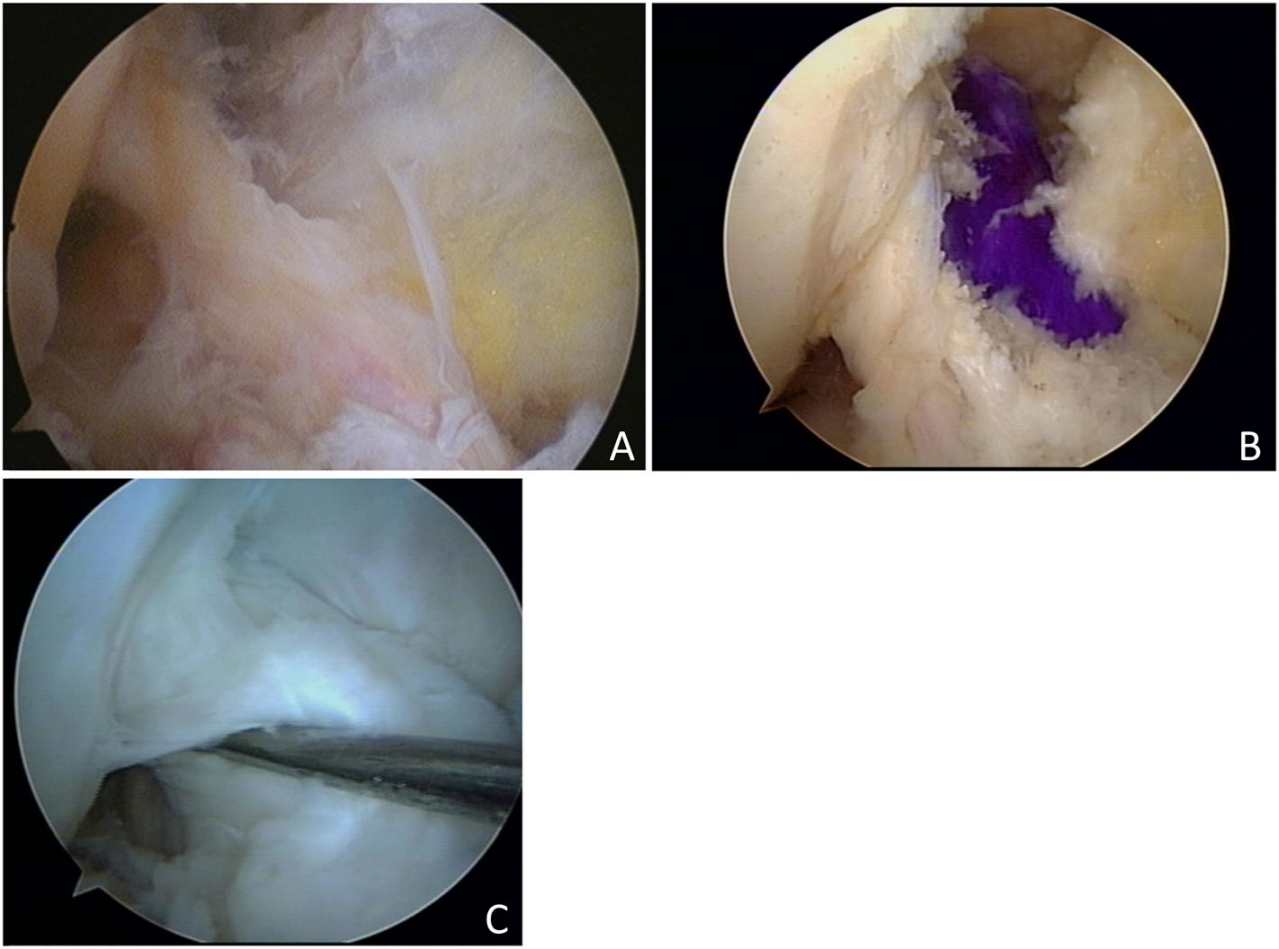
**a** Arthroscopic findings show an intact PL bundle which was well preserved and also had enough stability to be preserved. **b** AM bundle (*violet color* graft) was reconstructed and the intact PL bundle was still preserved during reconstruction. **c** Second-look arthroscopic findings in the same patient show good synovial coverage and distinguished AM and PL bundles

**Fig. 4:**
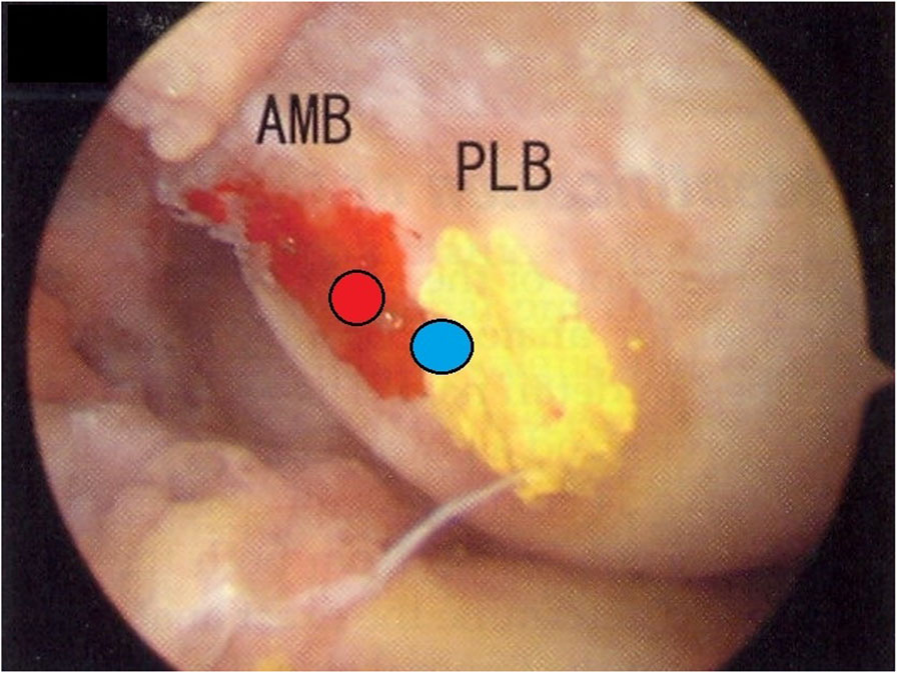
*Red dot* indicates center of femoral tunnel during AM bundle augmentation (Group 2), and *blue dot* indicates center of femoral tunnel during single-bundle ACL reconstruction (Group 1). *AMB* anteromedial bundle, *PLB* posterolateral bundle

A tibial tunnel was created at the center of the tibial attachment of the ACL in both groups. In the case of a single-bundle ACL reconstruction (Group 1)(Fig. 2a), we used an 8.0-mm or a 9.0-mm offset transtibial femoral tunnel guide to target the center of the ACL femoral attachment, and a femoral tunnel was placed in the 10 o’clock 30 min or 1 o’clock 30 min direction of the intercondylar notch through the transtibial femoral tunnel offset guide. In the other case of AM bundle augmentation operations (Group 2) (Fig. 3a, b), a femoral tunnel was placed in the center of the femoral attachment site of the AM bundle (Fig. 4).

The tibial and femoral tunnels were made slightly smaller than the expected diameter of the tunnels, and they were gradually enlarged to a diameter of about 7.5–9.0 mm by using a tunnel dilator. The grafted tendon barely passed through the tunnel and filled the tunnel, such that the joint fluid did not flow into the tunnel; this helped to heal the grafting ligament and surrounding bone tissue. The size of the tunnel diameter was determined by the diameter of the prepared grafts. Most of the tibial tunnel sizes were in the range between 7.5 and 9.0 mm in diameter. The femoral fixation was made using a bioabsorbable cross pin (RigidFix™), and the tibial area was secured with an absorbable interference screw in the tibial tunnel and additionally with a post tie using an anchor screw at the distal part of the tibial tunnel. Remnant tissue remaining at the tibial attachment of the ruptured ACL in Group 1 during surgery was preserved as much as possible, and if possible, we also sutured with grafted tendon using absorbable suture material. All operations were performed by a single surgeon.

### Postoperative rehabilitation

All patients followed the same postoperative rehabilitation program. From 3 days after operation, continuous passive motion was started. Until 4 weeks postoperatively, patients were restricted to partial weight bearing. Patients also were recommended to wear a knee brace until 3 months after surgery. From 3 months postoperatively, patients began to jog and perform quadriceps muscle strengthening exercises at a gymnasium.

### Assessment

The clinical results included the Lysholm score and Tegner activity score in preoperative check and final follow-up for the functional evaluation of the joints, and the Lachman test and pivot-shift test performed preoperatively and in the final follow-up for stability testing. In the event of screw irritation, post-tie screws used to secure transplant ligaments from the tibia were removed and second-look arthroscopy was done between 1 and 2 years after reconstruction. Based on the findings of second-look arthroscopy, we assessed the extent of synovial coverage in the grafted tendon (Figs. 2b, 3c). The same investigator, who was also the original surgeon for each case, assessed the extent of synovial coverage to ensure consistency of the measurements. The grafted ACLs were formally divided into three parts (proximal, middle, and distal thirds), the synovial coverage in each of the three parts was evaluated as a percentage, and the overall mean synovial coverage was calculated. To compare the areas of synovial coverage more accurately, the anterior, posterior, medial, and lateral sides were observed carefully, and a 70° arthroscope was used in areas with limited visibility [6, 14].

### Statistical analysis

The results of Group 1, who underwent the single-bundle ACL reconstruction, and Group 2, who had the AM bundle augmentation, were compared and statistically analyzed. All statistical data were expressed as mean ± standard deviation. As a statistical evaluation method, the IBM SPSS ver. 19.0 (IBM Corp., Armonk, NY, USA) statistics program was used. The clinical scores, Lysholm scores, and Tegner activity scores between the two groups were compared by the use of an unpaired *t* test, and the instability tests, such as the Lachman test and pivot-shift test, were compared by the Pearson chi-squared test and it was analyzed the results of Groups 1 and 2, and *p* < 0.05 was determined to be the statistically significant level.

## Results

The mean Lysholm score and Tegner activity score were 47.8 points and 3.1 points preoperatively, respectively, in Group 1, and improved postoperatively to 78.3 points and 7.2 points, respectively. Group 2 also improved to 89.1 points and 8.1 points, respectively, postoperatively, from 49.2 points and 3.2 points preoperatively, respectively (Lysholm score: *p* = 0.96, Tegner activity score: *p* = 0.351). At the final follow-up of 27.1 months, the mean score of Group 2 was superior to the mean score of Group 1, which was statistically significant (Lysholm score: *p* = 0.032, Tegner activity score: *p* = 0.046) at the final follow-up of 27.1 months (Table 3).

**Table 3:** Clinical results and synovialization

	Group 1 (SB group, *n* = 64)	Group 2 (Aug group, *n* = 21)	*p* value
Lysholm score			
Preoperative	47.8 ± 9.8	49.2 ± 8.3	0.96
Last follow-up (27.1 months)	78.3 ± 4.8	89.1 ± 4.2	0.032
Tegner activity score			
Preoperative	3.1 ± 1.2	3.2 ± 0.9	0.351
Last follow-up (27.1 months)	7.2 ± 1.3	8.1 ± 1.1	0.046
Synovial coverage (%) at 2nd-look A/S	61.4 (%) ± 4.3	93.1 (%) ± 3.2	0.008

*SB* single bundle, *Aug* anteromedial bundle augmentation, *A/S* arthroscopic surgery

In addition, in the Lachman test and the pivot-shift test, preoperative laxities were markedly improved at the final follow-up in both groups. However, these results were not statistically different in the comparison between Group 1 and Group 2 (Lachman test: *p* = 0.074, pivot-shift test: *p* = 0.131) (Table 4). Second-look arthroscopy showed that the percentage of synovial coverage was 61.4% and 93.1% in Group 1 and Group 2, respectively. The results were better in Group 2 than in Group 1 (*p* = 0.008) (Table 3). Cyclops lesions were found in one and two patients in Group 1 and Group 2, respectively; however, there were no accompanying clinical symptoms such as pain and limitation of extension. There were also hypertrophies of the grafted tendon in four patients, but there was no flexion contracture or extension block. All seven cases of cyclops lesions and hypertrophies were treated with a bipolar radiofrequency system (Arthrocare™, Arthrocare Corporation, Austin, TX, USA) during second-look arthroscopy.

**Table 4:** Results of anterior and rotatory stability testing

	Group 1 (SB group, *n* = 64)		Group 2 (Aug group, *n* = 21)		*p* value
	Preoperative	Last follow-up (27.1 months)	Preoperative	Last follow-up (27.1 months)	
Lachman test					0.074
–	6	54	3	19	
1+	22	10	9	2	
2+	28		7		
3+	8		2		
Pivot-shift					0.131
–	5	58	2	19	
1+	21	6	11	2	
2+	30		7		
3+	8		1		

*SB* single bundle, *Aug* anteromedial bundle augmentation

In our clinical series of patients, there were 37 cases (43.5%) of medial meniscus tear, 21 cases (24.7%) of medial collateral ligament injuries but not complete medial collateral ligament rupture, 17 cases (20.0%) of lateral meniscus tear, and others. The total number of associated injuries was 84 cases in 65 patients. Among the 37 medial meniscus tears, 28 cases were treated surgically, with subtotal medial meniscectomy (19 cases) and medial meniscus suture (9 cases). Among the 17 lateral meniscus tears, 15 cases were treated, with subtotal lateral meniscectomy (9 cases) and lateral meniscus suture (6 cases) (Table 2).

## Discussion

After ACL reconstruction, preservation of proprioception can maintain stable muscle reflex function around the knee joint. After the reconstruction, revascularization and synovialization of the graft tendon are also needed for prompt engraftment and prevention of retear [1, 2, 4, 5, 14–16]. Two methods are introduced as remnant preservation techniques. First, when the ACL is completely ruptured, we can preserve the remnant of the torn ACL at the tibial attachment site; it provides sufficient coverage of the grafted tendon at the entrance to the tibial tunnel [2, 3, 5, 15, 16]. Second, when the ACL is a partially ruptured AM bundle or PL bundle, we can perform a selective augmentation operation of the AM bundle or the PL bundle with preservation of the intact bundle [6, 8–11, 18, 19].

In this study, attempts were made to comparatively analyze the clinical outcomes of single-bundle ACL reconstruction when the ACL was completely ruptured (Group 1) and AM bundle augmentation with preservation of the PL bundle when the ACL was partially ruptured (Group 2). We hypothesized that the clinical outcomes and synovial coverage of Group 2 would be better than those of Group 1. According to our study, there were no significant differences in anterior stability between the groups, but clinical scores and synovial coverage were significantly better in Group 2 than in Group 1. Presumably, these results might support our hypothesis.

Several studies reported ACL reconstruction with a remnant preservation technique in which as much preservation as possible had superior results compared with the conventional ACL reconstruction technique [1, 2, 5, 8, 9, 14–16].

Nakamae et al. [7] reported that they did three types of ACL reconstruction: (1) AM bundle augmentation or PL bundle augmentation, (2) single-bundle reconstruction, and (3) double-bundle reconstruction, comparing and analyzing the three groups. There were no significant differences between the three groups in clinical results such as Lysholm score and pivot-shift test. There were some better results in the AM or PL augmentation group for synovial coverage from second-look arthroscopic examination, stability test, and test for proprioceptive function, but no statistical difference was observed.

Sonnery-Cottet et al. [9] introduced a new technique for ACL reconstruction such that four strands of hamstring tendon pass through the center of the remnant ACL, conserving as much remnant tissue as possible covering the graft tissue; this is known as single anteromedial bundle biologic augmentation (SAMBBA). This technique can help with synovialization because it helps preserve blood vessels and proprioceptive nerve endings in the remnant ligament tissue.

Matsushita et al. [20] compared PL bundle reconstruction in partial rupture of the ACL and double-bundle ACL reconstruction in complete rupture of the ACL. There were no differences in the instability test. The Lysholm score showed a better result in double-bundle ACL reconstruction, but the difference was not significant statistically. The authors stated that PL bundle augmentation is a comparable operation to double-bundle ACL reconstruction.

However, Park et al. [21] reported that there were no differences in both the anterior stability test and clinical results between the augmentation group and the double-bundle reconstruction group. There are some additional studies that showed no differences between augmentation and double-bundle reconstruction in the mechanical stability test.

Demirağ et al. [12] insisted that ACL reconstruction in partial rupture had an advantage concerning a prevention effect for tibial tunnel widening compared to conventional reconstruction.

Ochi et al. [1, 2] reported remnant preserving ACL reconstruction procedure which preserved as much of the mechanoreceptors in the remnant tissue of the torn ACL as possible; this procedure helps with regeneration of blood vessels and nerve endings to the graft tendon.

There have been many studies about revascularization. It was shown that if remnant tissue of the tibial attachment site was preserved during operation, neovascularity of the graft tendon and synovialization were aided because vascularity around the tibial attachment was maintained [8, 15–17].

Based on our results, the Lysholm score and Tegner activity score of Group 2 were superior to the scores for Group 1. The Lachman test and pivot-shift test scores of Group 2 were also better than those of Group 1. The area of synovial coverage at second-look arthroscopy of Group 2 was better than that for Group 1, and it was a statistically significant difference. As stated above, our results could show that Group 2 had better outcomes than Group 1 in view of functional evaluation and synovialization.

There are several limitations to this study. First, it was difficult to compare cases because the numbers of patients were different between Groups 1 and 2. Second, the interval of second-look arthroscopy of 15.6 months was too short from the time of reconstruction surgery. Third, the evaluation method of synovial coverage was subjective and estimated, and finally, there is a limit to the representation of each group because of short period  of follow-up at 27.1 months after surgery.

## Conclusion

AM bundle augmentation for ACL injury in which the PL bundle is preserved shows better clinical scores and synovial coverage than single-bundle ACL reconstruction for complete ACL rupture.

## Data Availability

Not applicable.

## Abbreviations

ACL: Anterior cruciate ligament
AM: Anteromedial
PL: Posterolateral
